# Hole-Doping-Induced Perpendicular Magnetic Anisotropy and High Curie Temperature in a CrSX (X = Cl, Br, I) Semiconductor Monolayer

**DOI:** 10.3390/nano13243105

**Published:** 2023-12-08

**Authors:** Ruilin Han, Xiaomin Xue, Yu Yan

**Affiliations:** 1 School of Physics and Electronic Engineering, Shanxi University, Taiyuan 030006, China; 2 Institute of Theoretical Physics, Shanxi University, Taiyuan 030006, China; 202122605012@email.sxu.edu.cn; 3 Key Laboratory of Physics and Technology for Advanced Batteries (Ministry of Education), Department of Physics, Jilin University, Changchun 130012, China; yanyu@jlu.edu.cn

**Keywords:** two-dimensional (2D) intrinsic ferromagnets, magnetic anisotropy energy, Curie temperature, first-principles calculations

## Abstract

A large perpendicular magnetic anisotropy and a high Curie temperature (T_C_) are crucial for the application of two-dimensional (2D) intrinsic ferromagnets to spintronic devices. Here, we investigated the electronic and magnetic properties of carrier-doped Van der Waals layered CrSX (X = Cl, Br, I) ferromagnets using first-principles calculations. It was found that hole doping can increase the magnitude of the magnetic anisotropy energy (MAE) and change the orientation of the easy magnetization axis at small doping amounts of 2.37 × 10^13^, 3.98 × 10^12^, and 3.33 × 10^12^/cm^2^ for CrSCl, CrSBr, and CrSI monolayers, respectively. The maximum values of the MAE reach 57, 133, and 1597 μeV/u.c. for the critical hole-doped CrSCl, CrSBr, and CrSI with spin orientation along the (001) direction, respectively. Furthermore, the Fermi energy level of lightly hole-doped CrSX (X = Cl, Br, I) moves into the spin-up valence band, leading to the CrSX (X = Cl, Br, I) magnetic semiconductor monolayer becoming first a half-metal and then a metal. In addition, the T_C_ can also be increased up to 305, 317, and 345 K for CrSCl, CrSBr, and CrSI monolayers at doping amounts of 5.94 × 10^14^, 5.78 × 10^14^, and 5.55 × 10^14^/cm^2^, respectively. These properties suggest that the hole-doping process can render 2D CrSX (X = Cl, Br, I) monolayers remarkable materials for application to electrically controlled spintronic devices.

## 1. Introduction

Since the discovery of monolayer CrI_3_ [1] and bilayer Cr_2_Ge_2_Te_6_ [2] in 2017, two-dimensional (2D) intrinsic ferromagnetic (FM) materials have attracted broad interest, both theoretically and experimentally, due to their exceptional physical properties and technological applications to high-performance spintronic devices. Van der Waals (vdW) transition-metal halides TMXY (TM = Cr, Mn; X = O, S, Se; Y = F, Cl, Br, I) are members of the 2D intrinsic FM material family; they have a rectangular spin lattice and have been reported to possess a remarkably higher Curie temperature (T_C_) than monolayer CrI_3_ and bilayer Cr_2_Ge_2_Te_6_ with a honeycomb spin lattice [3]. The outstanding features of vdW TMXY materials (TM = Cr, Mn; X = O, S, Se; Y = F, Cl, Br, I), including a weak interlayer interaction, a high T_C_, and a high ambient stability, make them suitable candidates as 2D magnetic materials for spintronic applications [4,5,6,7,8,9,10,11,12,13,14,15,16,17,18,19,20,21,22,23,24].

Among these transition metal halides TMXY (TM = Cr, Mn; X = O, S, Se; Y = F, Cl, Br, and I), CrOCl and CrSBr bulk and monolayers were obtained experimentally, and were reported to have an excellent air stability as well as be easily pliable and cleavable [25,26,27]. Previous studies have shown that the easy magnetization axis (EMA) of most 2D vdW CrXY (X = O, S, Se; Y = F, Cl, Br, I) monolayers lies in the plane, and the system exhibits triaxial magnetic anisotropy [5,6,7,8,9,10,11,12,14,15,21,23], which may provide more spin degrees of freedom for designing spintronic devices. However, the T_C_ of these materials is mostly still below room temperature, which hinders their application to practical spintronic devices. Moreover, perpendicular magnetic anisotropy (PMA) is an essential property of 2D intrinsic magnetic materials to achieve a high thermal stability and low critical current density for switching in next-generation high-density nonvolatile memories. Hence, it is highly desirable to achieve a large PMA in CrXY (X = O, S, Se; Y = F, Cl, Br, I) monolayers at above room temperature for their applications to high-performance spintronic devices.

Thus far, considerable efforts have been devoted to studying CrXY (X = O, S, Se; Y = F, Cl, Br, I) monolayers to enhance their T_C_ and obtain a large PMA through external methods. For example, researchers have predicted a remarkable enhancement of T_C_ in CrOF [6], CrSBr [13,15], CrSeBr [20], and CrSI [23,28] through doping. Additionally, several works have demonstrated that the T_C_ of CrOF, CrOCl, CrOBr, and CrSBr monolayers can be significantly enhanced by applying strain [4,12,29,30,31,32]. Furthermore, the application of strain and doping has also been reported to effectively manipulate the MAE of 2D CrOF, CrOCl, CrOBr, CrSCl, CrSBr, and CrSI, monolayers, as well as induce PMA [6,13,23,28,29,30,31,32]. In addition, vacancy engineering has also been predicted to be a feasible method for achieving PMA at room temperature [33]. Moreover, several theoretical and experimental works have reported that doping not only enhances the MAE and TC (accompanied by significant changes in the EMA direction), but also effectively alters the electronic structures and manipulates the magnetic properties of 2D materials, which could be beneficial for their applications [34,35,36,37,38,39,40,41,42,43,44,45,46,47,48,49,50,51]. The manipulation of the EMA to the out-of-plane direction and the enhancement of T_C_ to room temperature of CrSX (X = Cl, Br, I) monolayers via hole doping have been rarely reported.

In this work, we systematically investigate the effect of carrier doping on the electronic structure, magnetic properties, and T_C_ of CrSX (X = Cl, Br, I) monolayers using a combination of first-principles calculations and Monte Carlo (MC) simulations. Our calculations show that in contrast to the in-plane magnetic anisotropy of pristine CrSX (X = Cl, Br, I) monolayers, the EMA of hole-doped CrSX (X = Cl, Br, I) monolayers is in the out-of-plane direction. The enhanced MAE and the switching of the EMA due to hole doping can be ascribed to the competition between the positive contribution of the (*p_x_, p_y_*) orbitals and the negative contribution of the (*p_y_*, *p_z_*) orbitals of halogen atoms. Specifically, the T_C_ can be increased to room temperature for CrSCl, CrSBr, and CrSI monolayers at hole-doping amounts of 5.94 × 10^14^, 5.78 × 10^14^, and 5.55 × 10^14^/cm^2^, respectively. The ab initio molecular dynamics (AIMD) simulations and calculated phonon dispersion indicate that hole-doped CrSX (X = Cl, Br, I) monolayers are stable.

## 2. Computational Details

All the calculations were based on the first-principles density functional theory and were realized using the Vienna Ab initio Simulation Package (VASP) [52] with projector-augmented wave (PAW) potentials. The electron–ion and exchange correlations were considered using the generalized gradient approximation under the Perdew–Burke–Ernzerhof (PBE) functional [53,54]. Considering the strong correlation interaction of the Cr 3d electrons, the spin-polarized PBE + U method with *U*_eff_ = 3.0 eV was adopted based on recent studies [4,5,6,7,8,9,10,11,12,13,15,16,19,20,21,22]. The Heyd–Scuseria–Ernzerhof (HSE06) functional was also executed to confirm the accuracy of the electronic structures. All results were calculated with an energy cut-off of 500 eV. Then, 9×7×1 and 27×21×1 *k*-point samplings were utilized in the 2D Brillouin zone for geometry optimization and energy calculation, respectively. In the geometry optimization, the convergence criteria for the force and energy were set to $10^{-3}$ eV/Å and $10^{-8}$ eV, respectively. A 20-Å-thick vacuum layer was set in the z-direction to avoid spurious periodic interactions. The DFT-D3 method by Grimme was adopted to examine the effect of Van der Waals (vdW) interactions on monolayer CrSX (X = halogen atoms); such an effect was found to be negligible. The effect of spin–orbital coupling (SOC) was considered in the MAE calculations. A 3×3×1 supercell was constructed in the ab initio molecular dynamics (AIMD) simulation and phonon dispersion calculations. An 8.0 ps AIMD simulation was carried out with a time step of 1.0 fs at a constant temperature of 300 K. The phonon dispersion was calculated using the PHONOPY 2.14.0 software package [55]. The MC calculations were conducted to predict T_C_ using the VAMPIRE 5.0 binary packages [56] based on the anisotropic Heisenberg mode. We considered a larger 50 × 50 supercell with periodic boundary conditions in the specified temperature interval to eliminate the finite-size effects and completely thermalize the system to equilibrium with 120,000 scans starting from the ferromagnetic order; all statistics were obtained from the subsequent 720,000 scans. Simulations with carrier doping were treated by changing the number of total valence electrons in the unit cell with a compensating uniform background charge.

## 3. Results and Discussion

**Figure 1:**
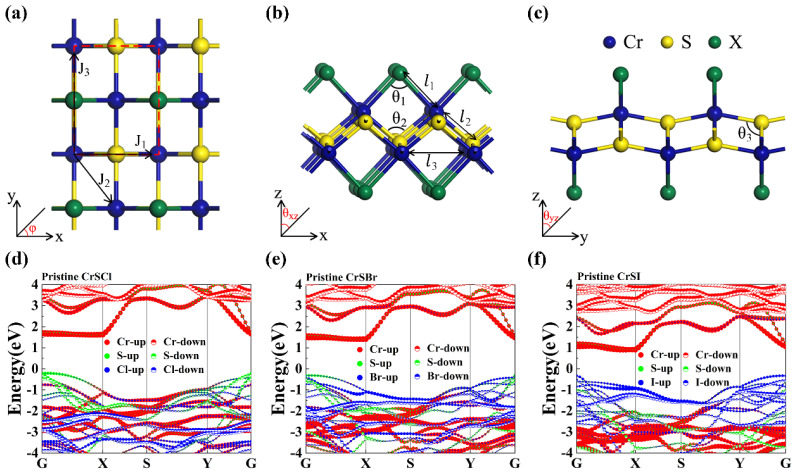
(**a**) Top and (**b**,**c**) side views of the atomic structure of a 2D CrSX (X = Cl, Br, I). Electronic band structure of the pristine (**d**) CrSCl, (**e**) CrSBr, and (**f**) CrSI monolayers with the HSE06 functional. The rectangle represents the unit cell. The labeled *J*_1_, *J*_2_, and *J*_3_ represent the first, second, and third nearest-neighbor exchange interaction between Cr and Cr atoms, respectively.

Figure 1a,b depicts the crystal structure of the CrSX (X = Cl, Br, I) monolayers, which has a rectangular orthorhombic lattice and belongs to the *Pmmn* space group. The red dotted lines in Figure 1a represent the unit cell (u.c.) of 2D CrSX (X = Cl, Br, I) monolayers, which contains two atoms of each element. Two Cr–S layers are sandwiched between the X (X = Cl, Br, I) layers. The optimized in-plane lattice constants, bond lengths, bond angles, and band gaps of the Janus CrSX (X = Cl, Br, I) monolayers are listed in Table S1, which agree well with the results of previous studies [5,8,12,15,16]. The corresponding electronic band structures of the CrSX (X = Cl, Br, I) monolayers are also calculated using the HSE06 functional and are displayed in Figure 1d–f, indicating that the CrSX (X = Cl, Br, I) monolayers are ferromagnetic semiconductors with band gaps of 1.78, 1.69, and 1.20 eV, respectively.

**Figure 2:**
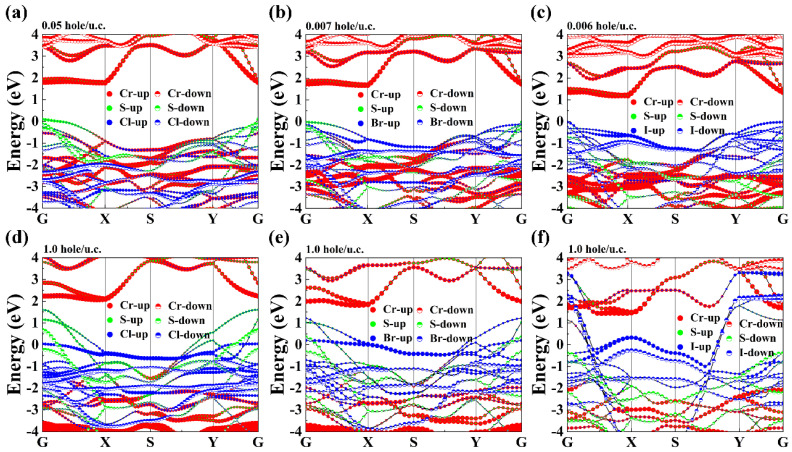
Electronic band structure of the 2D CrSX (X = Cl, Br, I) monolayers for different carrier doping densities. The doping densities are 0.05, 0.007, and 0.006 h/u.c. for the (**a**) CrSCl, (**b**) CrSBr, and (**c**) CrSI monolayers, respectively. (**d**–**f**) Scheme showing 1.0 hole/u.c. for 2D CrSX (X = Cl, Br, I) monolayers, respectively.

As is well known, carrier doping is a commonly used experimental technique for modifying the electronic, magnetic, and spin transport characteristics of 2D materials. Experimentally, a charge density of 2D materials can be injected to be as high as 10^15^ cm^−2^, which can be achieved through the application of a gate voltage technique [57,58,59,60]. In our work, 0.1 hole (about 10^13^ cm^−2^), 0.2 hole (about 10^14^ cm^−2^), and 0.7 hole (about 10^14^ cm^−2^) or electrons were incorporated into the monolayer CrSX (X = halogen atoms); these doping concentrations are on the same order of magnitude as the experimental limit (about 10^15^ cm^−2^). Therefore, we incorporated from 0 to 1.0 holes (equivalent to 5.94 × 10^14^, 5.78 × 10^14^, and 5.55 × 10^14^/cm^2^ for CrSCl, CrSBr, and CrSI, respectively) in units of concentration for 2D CrSX (X = Cl, Br, I). The band structure of lightly or 1.0-hole-doped 2D CrSX (X = Cl, Br, I) was calculated using the HSE06 functional, and the results are shown in Figure 2a–f. The introduction of more holes causes the Fermi level to gradually shift downward, which results in the transformation of CrSX (X = Cl, Br, I) from a semiconductor to a half-metal and then to a metal. Similar to the pristine systems, during hole doping, the valence band states near the Fermi level are primarily contributed to by the *p* orbitals of the non-metallic S and X atoms, while the conduction band is largely generated by the *d* orbitals of the Cr atoms. We observe a gradual increase in hybridization between the *p* orbitals of sulfur and the *p* orbitals of halogen with increasing hole doping density.

**Figure 3:**
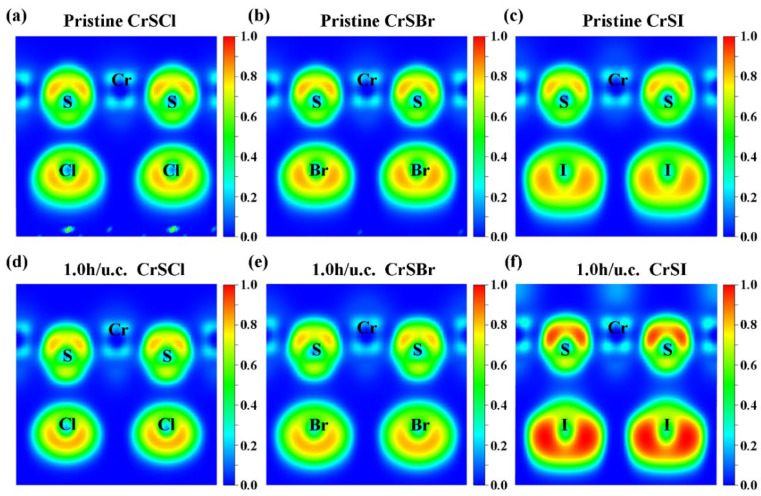
Calculated electron localization function and its projection onto the (100) plane for the pristine (**a**–**c**) CrSX (X = Cl, Br, I) monolayers and 1.0-hole-doped (**d**) CrSCl, (**e**) CrSBr, and (**f**) CrSI monolayers, respectively.

To explore the bonding properties both before and after hole doping more accurately, we calculated the electron localization function (ELF) for the hole-doped 2D CrS*X* (*X* = Cl, Br, I). We then projected the ELF onto the (100) plane, as depicted in Figure 3, where 0 and 1 represent fully localized and fully delocalized electrons, respectively. The data show that the regions between the Cr and S atoms and between the Cr and *X* (*X* = Cl, Br, I) atoms have lower ELF values, suggesting a clear ionic bonding nature. We can also observe that the distribution of higher ELF values in the systems is apparent at the *X* (*X* = Cl, Br, I) sites, which can be explained in terms of the higher electronegativity of the X atoms compared with that of the Cr and S atoms. Moreover, the spin-polarized densities are plotted in Figure S1 to quantitatively analyze the spin orientation. As observed in Figure S1, there are different spin orientations between the Cr, S, and X atoms; this is also demonstrated by the calculated magnetic moments of the Cr, S, and X (X = Cl, Br, I) atoms, which are plotted in Figure S2.

Next, in order to determine the preferred magnetic ground state of hole-doped 2D CrSCl, CrSBr, and CrSI, we calculated the total energy of the spin-polarized ferromagnetic (FM) order and three feasible types of spin-polarized antiferromagnetic (AFM) order for all three monolayers, as illustrated in Figure S3. Additionally, four different magnetic configurations were also constructed to calculate the exchange-coupling parameters J_1,_ J_2_, and J_3_. According to the classical Heisenberg Monte Carlo model, the spin Hamiltonian model is defined as follows:

$$H=-J\sum_{j,k}S_{j}\cdot S_{k}-A\sum_{j}{\left(S_{j}^{z}\right)}^{2}$$

Then, the exchange coupling constant, J, can be calculated as follows:

(1) $$E_{FM}=E_{0}-\left(4J_{1}+2J_{2}+2J_{3}\right)S^{2}-AS^{2}$$

(2) $$E_{AFM1}=E_{0}-(-4J_{1}+2J_{2}+2J_{3})S^{2}-AS^{2}$$

(3) $$E_{AFM2}=E_{0}-\left(-2J_{2}-2J_{3}\right)S^{2}-AS^{2}$$

(4) $$E_{AFM3}=E_{0}-\left(-2J_{2}+2J_{3}\right)S^{2}-AS^{2}$$

**Figure 4:**
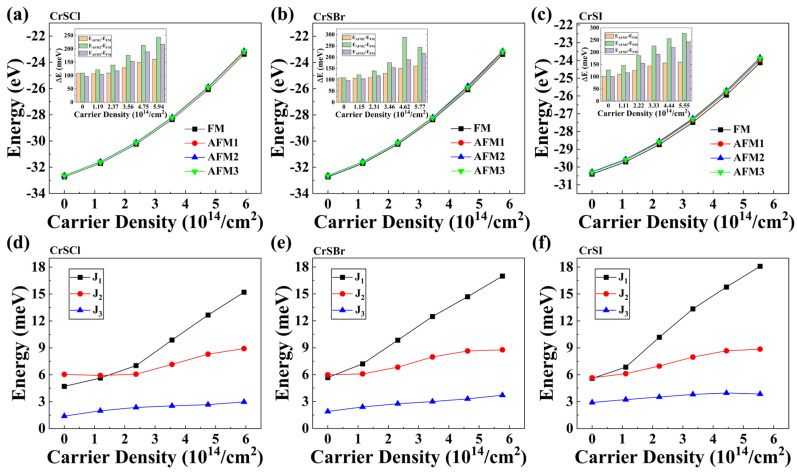
FM, AFM1, AFM2, and AFM3 order energies as a function of the hole doping density for 2D (**a**) CrSCl, (**b**) CrSBr, and (**c**) CrSI. Exchange coupling parameters J_1_, J_2_, and J_3_ as a function of the hole doping density for 2D (**d**) CrSCl, (**e**) CrSBr, and (**f**) CrSI.

Here, $S_{j}$, $S_{k}$, *A*, and $S_{j}^{z}$ denote the exchange coupling parameter, spin vectors of the *j* and *k* sites, anisotropy energy, and spin along the EMA, respectively. *A* is expressed as $A=\left[Emax\left(axis\right)-Eeasy\left(axis\right)\right]/{\left|S\right|}^{2}.$ The calculated anisotropy energy *A* for the pristine and hole-doped CrSX (X = halogen atoms) are shown in Table S2. The calculated exchange-coupling parameters *J*_1_, *J*_2_, and *J*_3_ for the hole-doping case are displayed in Figure 4d–f. One can see that as the hole-doping concentration increases, Δ*E*_1_, Δ*E*_2_, Δ*E*_3_, and the exchange coupling parameters of the three hole-doped CrSX (X = Cl, Br, I) increase and are positive, indicating that hole doping promotes the establishment of an FM ground state. To gain a deeper understanding of the magnetic coupling mechanism of a hole-doped CrSX (X = Cl, Br, I), we calculated the nearest Cr–Cr distance, d. Figure S3 shows that such a distance decreases slightly monotonically with increasing hole doping density, but it always remains large, even for a 1.0-hole-doped CrSX (X = Cl, Br, I). In addition, the average cation–anion–cation bond angles (see Figure 1b and Figure S4) in the hole-doped CrSX (X = Cl, Br, I) monolayers are closer to 90°, confirming that the FM ground state is more stable for the hole-doped CrSX (X = Cl, Br, I) monolayers according to the Goodenough–Kanamori–Anderson rules [61,62,63]. Thus, the competition of the direct exchange interaction between the nearest-neighbor transition metal atoms and the TM–S/X–TM super-exchange interaction dictates the magnetic ground states of the hole-doped 2D CrSX (X = Cl, Br, I). Furthermore, due to the increase in ΔE, the J_1_, J_2_, and J_3_ obtained for the 1.0-hole-doped CrSX (X = Cl, Br, I) monolayers are larger than those of the pristine CrSX (X = Cl, Br, I) monolayers. In other words, with the increase in hole doping concentration, the three magnetic pair interactions of the hole-doped monolayer CrSX (X = Cl, Br, I) shown in Figure 4d–f become gradually stronger; especially, J_1_ is significantly enhanced, which indicates that the ferromagnetic coupling of the hole-doped system is significantly enhanced. This leads to a significant increase in the TC of the monolayer CrSX (X = Cl, Br, I). From the band structure with the HSE06 functional in Figure 2 and the exchange coupling parameters as a function of the hole-doping density in Figure 4, we find that charge doping can introduce mobile carriers into the CrSX (X = Cl, Br, I) monolayers to modulate the FM exchange of the newly formed half-metallic or metallic state, leading to an enhanced FM stability of the hole-doped 2D CrSX (X = Cl, Br, I).

**Figure 5:**
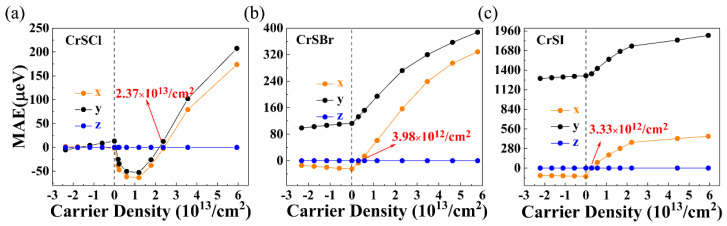
Carrier density dependence of the MAE for the 2D (**a**) CrSCl, (**b**) CrSBr, and (**c**) CrSI monolayers.

It is known that the PMA improves the storage density of spintronic devices. However, the EMA of the pristine CrSX (X = Cl, Br, I) monolayers lies in the plane, and it needs to be switched to PMA to achieve a high-density storage for the CrSX (X = Cl, Br, I) monolayers. Here, the MAE of the pristine and hole-doped 2D CrSX (X = Cl, Br, I) is also investigated including the SOC. The obtained MAE (E_(100/010)_ − E_(001)_) values for the pristine CrSCl, CrSBr, and CrSI are −1.85 μeV per u.c./13.20 μeV per u.c., −24.75 μeV per u.c./112.45 μeV per u.c., and −90.15 μeV per u.c./1318.75 μeV/u.c., respectively, indicating that the anisotropy is in the x–y plane and the EMA of the pristine CrSCl, CrSBr, and CrSI is along the in-plane (100) direction. Then, the variation in the MAE in the range from 0.04 to 0.18 hole per u.c. for 2D CrSX (X = Cl, Br, I) including the SOC, as shown in Figure 5a–c. From Figure 5, it was found that the EMA of an electron-doped CrSX (X = Cl, Br, I) remains in the *x–y* plane, and the (100) direction is the most energetically favorable among the three considered spin orientations. By contrast, the orientation of the EMA for CrSCl, CrSBr, and CrSI can be more effectively switched via hole-doping than electron doping. The critical points for the out-of-plane magnetization are 2.37 × 10^13^, 3.98 × 10^12^, and 3.33 × 10^12^/cm^2^ for the CrSCl, CrSBr, and CrSI monolayers, where the critical hole doping concentration of CrSBr is consistent with previous work [13]. As can be seen in Table 1, the (001) direction of the critical hole-doped 2D CrSCl/CrSBr/CrSI has the lowest MAE, which is 38.05/8.5/112.72, 57.1/133.2/1596.55, 24.7/66/725.1, 15.45/3.5/5.1, 47.75/7.9/843.3, and 28.5/46.65/496.2 μeV per u.c. in energy among those of the given spin orientation along (100), (010), (011), (101), (110) and (111) directions, respectively, both indicating that the EMA is along the z-direction.

For 2D CrSX (X = Cl, Br, I) monolayers with an orthogonal crystal symmetry, the corresponding angular-dependent MAE can be written as: [14]

*MAE (θ, φ) = K*_1_*sin*^2^*θ + K*_2_*sin*^2^*θsin*^2^*φ + K*_3_*sin*^4^*θ + K*_4_*sin*^4^*θsin*^2^*φ + K*_5_*sin*^4^*θsin*^4^*φ* (5)

**Figure 6:**
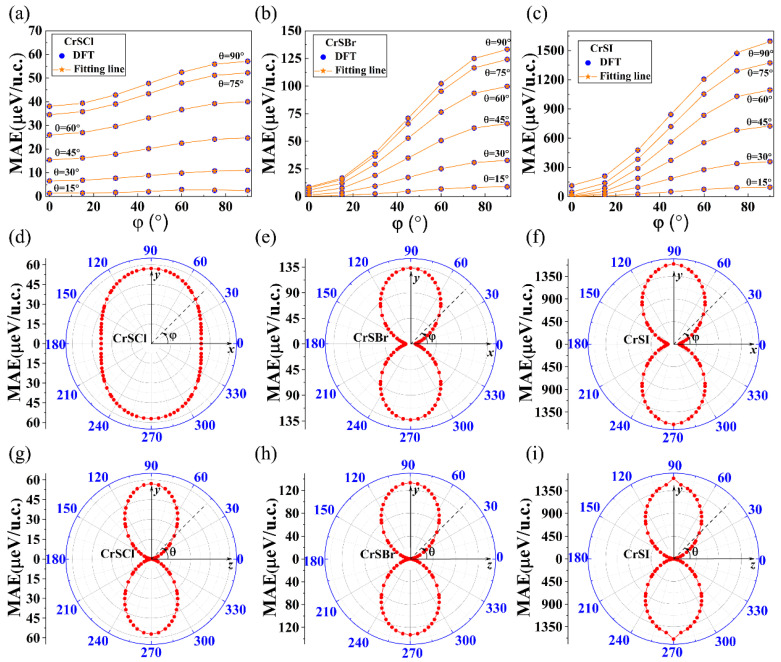
Fitting results of the MAE for the given spin orientations of hole-doped (**a**) CrSCl, (**b**) CrSBr, and (**c**) CrSI. Angular-dependent MAE with the spin orientations in the *x–y* planes for hole-doped (**d**) CrSCl, (**e**) CrSBr, and (**f**) CrSI. Angular-dependent MAE with the spin orientations in the *y–z* planes for hole-doped (**g**) CrSCl, (**h**) CrSBr, and (**i**) CrSI.

**Figure 7:**
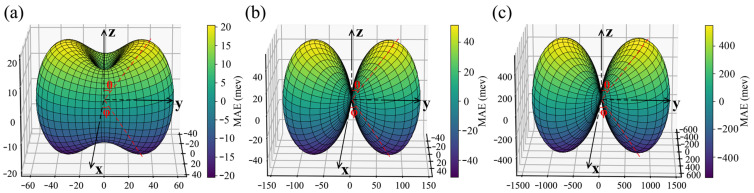
Angular-dependent energy (μeV/u.c.) for the critical hole-doped (**a**) CrSCl, (**b**) CrSBr, and (**c**) CrSI monolayers with the spin orientation lying in the whole space.

Here, *K_i_* represents the anisotropy constants and (*θ*, *φ*) represent the polar angle and azimuth angle in spherical coordinates. The magnetic anisotropy constants *K_i_* were obtained by fitting the energy data shown in Figure 6a–c for the hole-doped CrSX (X = Cl, Br, I) monolayers. It can be seen from Table 2 that the preferred magnetization direction for all three hole-doped CrSX monolayers (X = Cl, Br, I) is along the z-direction due to K_1_ and K_2_ being positive, and K_3_, K_4_, and K_5_ being non-zero. The maximum values of the MAE reach 57, 133, and 1597 μeV/u.c. for the hole-doped CrSCl, CrSBr, and CrSI at (*θ*, *φ*) = (90°, 90°), as plotted in Figure 6d–i. It can also be seen from Figure 6f,i that when θ is equal to 0° or 270°, the MAE of CrSI possesses the largest value of 1473 μeV/u.c. From the top view of the crystal structure shown in Figure 1b,c, it was found that the I–Cr bond length $l_{1}$ corresponds to an angle of 90°. Combined with the bond length information of the critical hole-doped CrSX (X = Cl, Br, I) listed in Table S3, it was found that the $l_{1}$ distance is maximized in the CrSI system. Therefore, I is subjected to the lowest repulsion when θ is equal to 90°, which means that when θ is equal to 90°, the MAE of I has the largest value. The corresponding energy of the hole-doped CrSX (X = Cl, Br, I) with the spin orientation being in the whole space is calculated and presented in Figure 7a–c, which further demonstrates that the hole-doped CrSX (X = Cl, Br, I) monolayers have a large MAE with the EMA along the *z*-axis.

**Figure 8:**
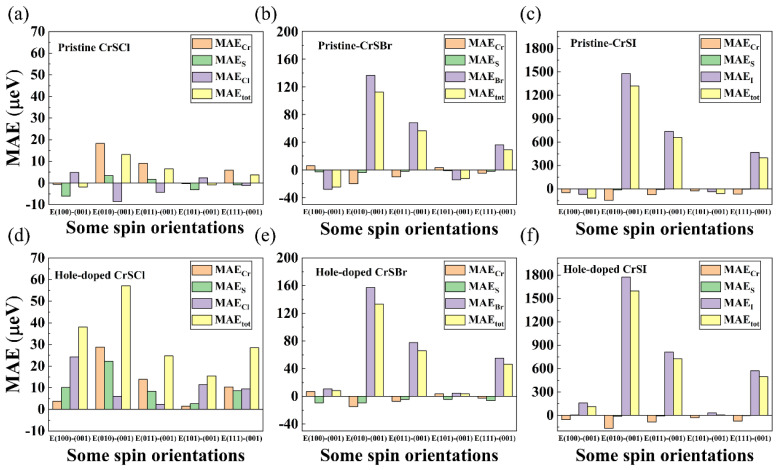
Atom-resolved MAE for the pristine (**a**) CrSCl, (**b**) CrSBr, and (**c**) CrSI and critical hole-doped (**d**) CrSCl, (**e**) CrSBr, and (**f**) CrSI.

To clarify the origin of the MAEs, we calculated the atom-resolved MAE of the pristine and critical hole-doped CrSX (X = Cl, Br, I), as illustrated in Figure 8. The results show that the MAEs are sensitive to the introduction of holes in the CrSX (X = Cl, Br, I) monolayers, which can increase the magnitude of the MAE and change the direction of the EMA. More importantly, the non-metallic atoms contribute the most to the total MAE in the hole-doped CrSX (X = Cl, Br, I) monolayers.

To explain the roles of non-metallic atoms in the total MAE of the hole-doped CrSX (X = Cl, Br, I) monolayers, we express the MAE based on the second-order perturbation theory as follows [64]:

(6) $$\begin{array}{cc} MAE\left(\theta ,\varphi \right)=\xi^{2} & \sum_{o^{+},u^{+}}\frac{{\left|\left\langle o^{+}|{\hat{L}}_{z}|u^{+}\right\rangle \right|}^{2}-{{\left|\left\langle o^{+}|{\hat{L}}_{\theta ,\varphi }|u^{+}\right\rangle \right|}^{2}}^{2}}{\varepsilon_{u}^{+}-\varepsilon_{o}^{-}}\\ & +\xi^{2}\sum_{o^{-},u^{+}}\frac{{\left|\left\langle o^{-}|{\hat{L}}_{\theta ,\varphi }|u^{+}\right\rangle \right|}^{2}-{\left|\left\langle o^{-}|{\hat{L}}_{z}|u^{+}\right\rangle \right|}^{2}}{\varepsilon_{u}^{+}-\varepsilon_{o}^{-}} \end{array}$$

Here, ξ, ${\hat{L}}_{z}$, $\varepsilon_{u}^{+}$, and $\varepsilon_{o}^{-}$ denote the SOC constant, angular momentum operators, and energy levels of the unoccupied spin-up states and occupied spin-down states, respectively. The summation in Equation (6) is, on the one hand, related to the energy difference between the occupied and unoccupied states in the denominator. On the other hand, it depends on the square difference of the matrix elements in the molecule. The difference in energy between the occupied and unoccupied states is mainly determined by the electronic states near the Fermi level. Therefore, we only consider the MAE in the same spin-up states because the larger band gap makes a negligible contribution to the total MAE in other spin states. We take the (100) direction as an example to probe the contribution of non-metallic atoms to the total PMA, and the orbital-resolved MAE of the pristine and critical hole-doped CrSX (X = Cl, Br, I) are shown in Figures S5–S7. For the CrSCl monolayer, the atomic numbers of the various atoms in the constituent system are relatively close, so the change in Cl to change the MAE is not very clear. We focus on the critical hole-doped CrSBr and CrSI shown in Figures S5 and S6; the comparison with CrSX (X = Br, I) shows a significant increase in the total MAE, and it changes from negative to positive as X changes from Br to I. We also found that the SOC of the heavy Br/I atoms has a significant effect on the total PMA, especially for the I atoms. Our results show that the PMA of monolayers CrSBr and CrSI is mainly caused by the spin-polarized *p* orbitals of the non-metallic Br and I atoms, while the contribution of the magnetic Cr atoms to the total PMA is negligible, as shown in Figure 8. The reason is that the MAE originates from the SOC term, and the SOC increases with the increasing atomic number of the X atom (from Br to I). As can be seen from Figures S6 and S7, the competition between the positive contribution of the (*p_x_*, *p_y_*) orbitals of the Br/I atoms and the negative contribution of the (*p_y_*, *p_z_*) orbitals leads to a positive total MAE. The corresponding high-orbital-resolved DOS of *p_x_* and *p_y_* in the valence bands of the critical hole-doped CrSX (X = Cl, Br, I) also confirms this phenomenon. Furthermore, the phonon spectra and AIMD simulations of the critical hole-doped 2D CrSX (X = Cl, Br, I) are shown in Figure S8. The inserts in Figure S8d–f show the top and side views of the 3 × 3 × 1 supercell structure at 4ps and 8ps at the end of molecular–dynamics simulation. The absence of imaginary phonon modes and negligible energy fluctuations at 300 K confirms that hole-doped 2D CrSX (X = Cl, Br, I) monolayers exhibit good kinetic and thermal stabilities.

**Figure 9:**
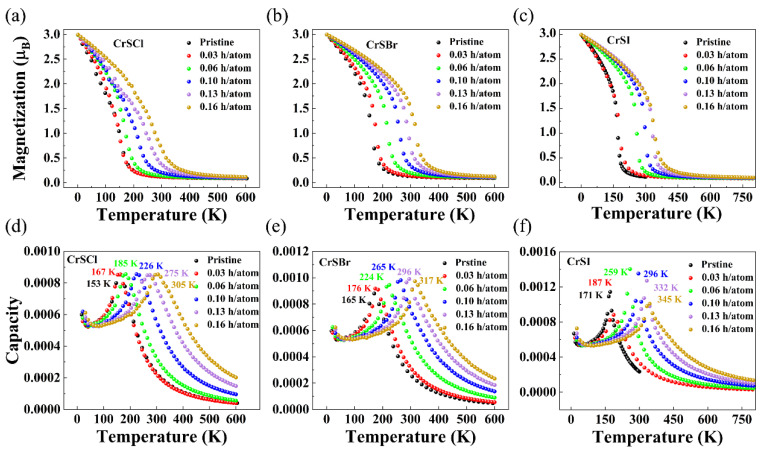
(**a**–**c**) Magnetic moment and (**d**–**f**) Monte Carlo-simulated specific heat (C_V_) as a function of temperature for 2D CrSX (X = Cl, Br, I) with various hole doping concentrations.

Hole doping results in a stronger FM exchange interaction and, as a result, a higher T_C_ for the hole-doped CrSX (X = Cl, Br, I) monolayers. Thus, MC simulations based on the anisotropic Heisenberg model were performed to determine the T_C_ of hole-doped 2D CrSX (X = Cl, Br, I). As illustrated in Figure 9, the variation in the magnetic moment and specific heat with temperature for various hole doping concentrations was calculated via MC simulations. According to the MC simulations, the T_C_ can be obtained either by reducing the magnetic moment to zero or by locating the peak position of the specific heat C_v_. The estimated T_C_ of 153, 165, and 171 K for the pristine 2D CrSX (X = Cl, Br, I) monolayers agrees with previous theoretical predictions and experimental results [4,7,11,12,14]. Figure 9 also shows the variation in T_C_ for 2D CrSX (X = Cl, Br, I) at various hole-doping concentrations. It can be seen that T_C_ increases monotonically to room temperature at a doping concentration of 1.0 hole/u.c. (equivalent to 5.94 × 10^14^, 5.78 × 10^14^, and 5.55 × 10^14^ cm^−2^) for the CrSCl, CrSBr, and CrSI monolayers, suggesting that 2D CrSX (X = Cl, Br, I) monolayers could be potential spintronic materials with electric-field-tunable magnetism.

## 4. Conclusions

In conclusion, we used first-principles calculations to predict a hole-doped 2D CrSX (X = Cl, Br, I) with high kinetic and thermal stability, strong PMA, and high T_C_. With the increase in hole doping concentration, a semiconductor to half-metal to metal transition can be triggered in 2D CrSX (X = Cl, Br, I). External hole doping causes a charge transfer and results in the Fermi energy level moving upward into the valence band, further enhancing the FM exchange and increasing the magnetic transition temperature. The competition between the positive contribution of the (*p_x_*, *p_y_*) orbitals and the negative contribution of the (*p_y_*, *p_z_*) orbitals for heavy atoms leads to a giant PMA enhancement for hole-doped 2D CrSX (X = Cl, Br, I). These outstanding properties make 2D CrSX (X = Cl, Br, I) a potential spintronic material with electric-field-tunable magnetism.

## Figures and Tables

**Table 1:** The MAE (μeV) of the critical hole-doped CrSX (X = Cl, Br, I) of the given spin orientation along (100), (010), (011), (101), (110), and (111) directions, respectively. The energy with the spin orientation along the (001) direction is a reference.

	(001)	(100)	(010)	(011)	(101)	(110)	(111)
**CrSCl**	0	38.05	57.1	24.7	15.45	47.75	28.5
**CrSBr**	0	8.5	133.2	66	3.5	7.9	46.65
**CrSI**	0	112.75	1596.55	725.1	5.1	843.3	496.2

**Table 2:** Constants of magnetic anisotropy *K_i_* (μeV) of the critical hole-doped CrSX (X = Cl, Br, I) monolayer.

	K_1_	K_2_	K_3_	K_4_	K_5_
**CrSCl**	23.75	17.95	14.3	1.8	−0.7
**CrSBr**	5.5	126.2	3	1.8	−0.2
**CrSI**	88.1	1215.75	24.65	222.65	45.4

## Data Availability

The data that support the findings of this study are available upon reasonable request from the authors.

## Supplementary Materials

The following supporting information can be downloaded at: https://www.mdpi.com/article/10.3390/nano13243105/s1, Table S1. Calculated in-plane lattice constants, bond lengths, bond angles and band gaps of the Janus CrSX (X = Cl, Br, I) monolayers. Table S2. Calculated anisotropy energy parameter A for the pristine and 1.0 hole-doping CrSX (X = halogen atoms). Table S3. Calculated in-plane lattice constants, bond lengths, bond angles and band gaps of the critical hole-doped CrSX (X = Cl, Br, I) monolayers. Figure S1. The spin polarized charge density for the pristine (a)–(c) CrSX (X = Cl, Br, I) monolayers and 1.0 hole-doped (d) CrSCl, (e) CrSBr, and (f) CrSI monolayers, respectively. The spin-up density is shown in yellow and the spin-down density is shown in blue, respectively. The iso-surface value is set to 0.005 eÅ^−3^. Figure S2. Magnetic moment as a function of the hole-doping density for 2D (a) CrSCl, (b) CrSBr, and (c) CrSI. Figure S3. Schematic illustrations of four feasible types of magnetic configurations are considered for 2D CrSX (X = Cl, Br, I): (a) FM, (b) AFM1, and (c) AFM2, and (d) AFM3. Red and blue arrows denote spin up and spin down directions, respectively. Figure S4. Hole-doping dependence of the nearest Cr–Cr distance and the average bond angle of cation-anion-cation for the 2D (a) CrSCl, (b) CrSBr, and (c) CrSI monolayer. Figure S5. Orbital-projected contribution to MAE for (a) Cr, (b) S, and (c) Cl atom for the pristine CrSCl monolayer with spin orientation along (100) direction. Orbital-projected contribution to MAE for (d) Cr, (e) S, and (f) Cl atom for the hole-doped CrSCl monolayer with spin orientation along (100) direction. Figure S6. Orbital-projected contribution to MAE for (a) Cr, (b) S, and (c) Br atom for the pristine CrSBr monolayer with spin orientation along (100) direction. Orbital-projected contribution to MAE for (d) Cr, (e) S, and (f) Br atom for the hole-doped CrSBr monolayer with spin orientation along (100) direction. Figure S7. Orbital-projected contribution to MAE for (a) Cr, (b) S, and (c) Iatom for the pristine CrSCl monolayer with spin orientation along (100) direction. Orbital-projected contribution to MAE for (d) Cr, (e) S, and (f) I atom for the hole-doped CrSI monolayer with spin orientation along (100) direction. Figure S8. Phonon spectra of hole-doped 2D (a) CrSCl, (b) CrSBr, and (c) CrSI. AIMD simulations of hole-doped 2D (d) CrSCl, (e) CrSBr, and (f) CrSI at 300 K.
