# Supplementary material for: Hole-Doping-Induced Perpendicular Magnetic Anisotropy and High Curie Temperature in a CrSX (X = Cl, Br, I) Semiconductor Monolayer
Source: Nanomaterials (Basel). 2023 Dec 8;13(24):3105. doi: 10.3390/nano13243105 (PMC10745588; doi:10.3390/nano13243105)
Supplement: Supplementary file 1 [file nanomaterials-13-03105-s001.zip › nanomaterials-2726336-supplementary.pdf]

Supplemental Material Information

"Hole-doping-induced Perpendicular Magnetic Anisotropy and Room-Temperature Ferromagnetism in Monolayer CrSX (X=Cl, Br, I)"

Ruilin Han,*[a] Xiaomin Xue,[b] and Yu Yan[c]

[a] *School of Physics and Electronic Engineering, Shanxi University, Taiyuan 030006, China*

[b] *Institute of Theoretical Physics, Shanxi University, Taiyuan, 030006, China*

[c] *Key Laboratory of Physics and Technology for Advanced Batteries (Ministry of Education), Department of Physics, Jilin University, Changchun 130012, China*

*E-mail: hanruilin0116@sxu.edu.cn

Table S1. Calculated in-plane lattice constants, bond lengths, bond angles and band gaps of the Janus CrSX (X=Cl, Br, I) monolayers.

Pristine	a(Å)	b(Å)	θ_1(°)	θ_2(°)	θ_3(°)	l_1(Å)	l_2(Å)	l_3(Å)	band-gap(eV)
CrSCl	3.48	4.83	93.21	92.44	97.03	2.39	2.41	3.48	1.78
CrSBr	3.58	4.82	89.61	95.32	96.99	2.54	2.43	3.58	1.69
CrSI	3.75	4.81	86.02	99.71	96.96	2.75	2.45	3.75	1.20

Table S2. Calculated anisotropy energy parameter A for the pristine and 1.0 hole-doping CrSX (X=halogen atoms).

**anisotropy energy parameter**	**CrSCl**		**CrSBr**		**CrSI**	
	Pristine	1.0 hole	Pristine	1.0 hole	Pristine	1.0 hole
**A (μeV)**	6.69	35.44	60.98	565.64	638.51	474.62

Table S3. Calculated in-plane lattice constants, bond lengths, bond angles and band gaps of the critical hole-doped CrSX (X = Cl, Br, I) monolayers.

critical hole-doped	a(Å)	b(Å)	θ_1(°)	θ_2(°)	θ_3(°)	l_1(Å)	l_2(Å)	l_3(Å)
CrSCl (0.05h)	3.481	4.822	93.433	92.367	97.452	2.390	2.412	3.481
CrSBr (0.007h)	3.584	4.822	89.654	95.282	97.034	2.542	2.425	3.584
CrSI (0.006h)	3.749	4.808	86.023	99.714	96.964	2.748	2.452	3.749

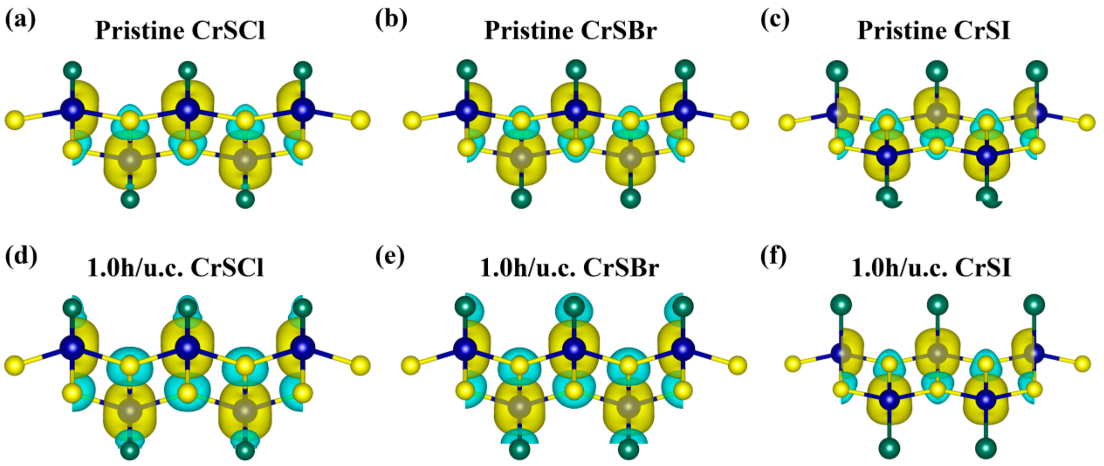

Figure S1. The spin polarized charge density for the pristine (a)–(c) CrSX (X = Cl, Br, I) monolayers and 1.0 hole-doped (d) CrSCl, (e) CrSBr, and (f) CrSI monolayers, respectively. The spin-up density is shown in yellow and the spin-down density is shown in blue, respectively.The iso-surface value is set to 0.005 eÅ^{-3}.

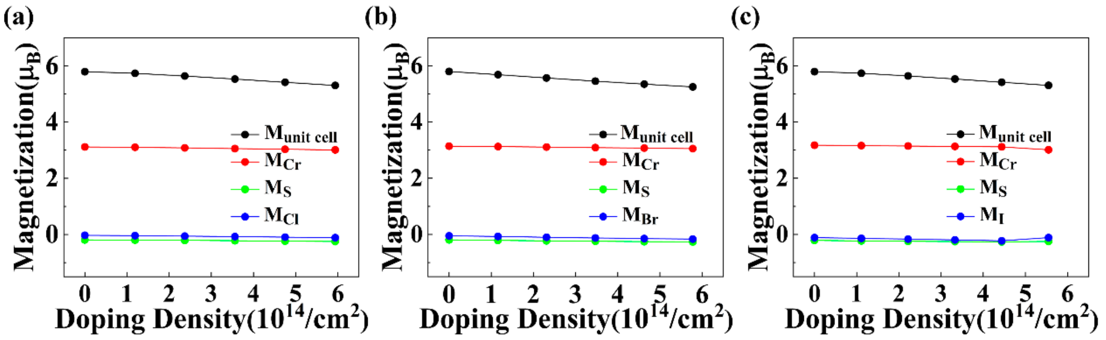

Figure S2. Magnetic moment as a function of the hole-doping density for 2D (a) CrSCl, (b) CrSBr, and (c) CrSI.

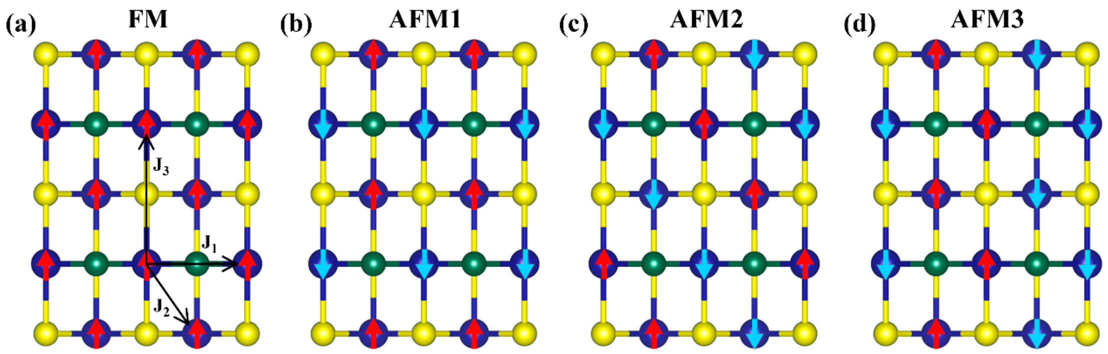

Figure S3. Schematic illustrations of four feasible types of magnetic configurations are considered for 2D CrSX (X=Cl, Br, I): (a) FM, (b) AFM1, and (c) AFM2, and (d) AFM3. Red and blue arrows denote spin up and spin down directions, respectively.

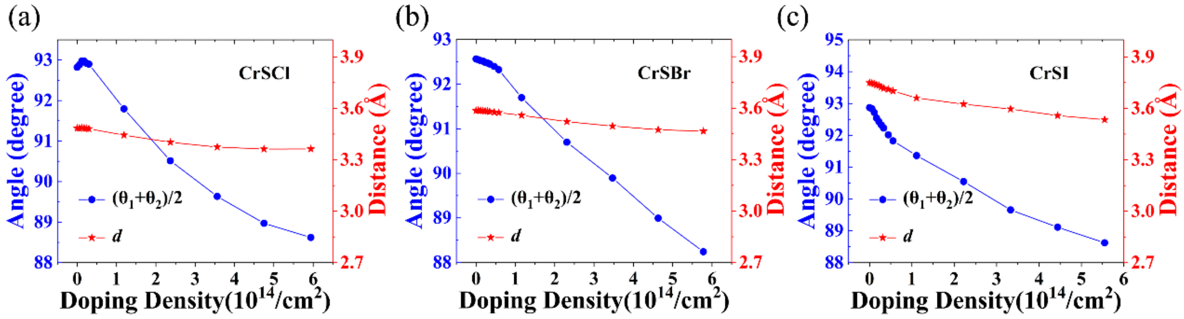

Figure S4. Hole-doping dependence of the nearest Cr–Cr distance and the average bond angle of cation-anion-cation for the 2D (a) CrSCl, (b) CrSBr, and (c) CrSI monolayer.

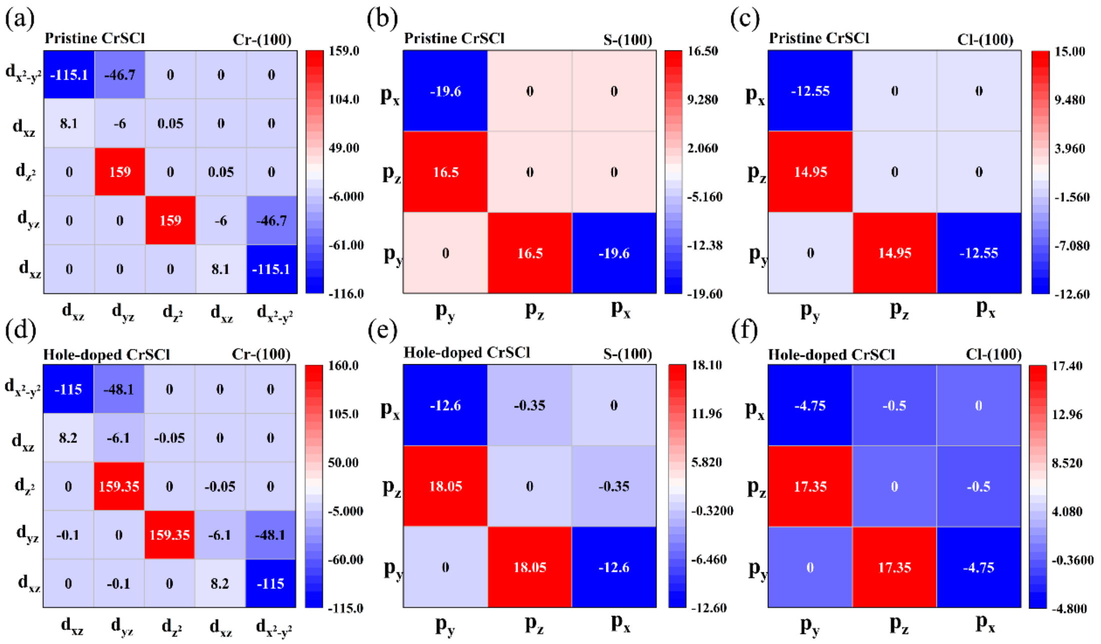

Figure S5. Orbital-projected contribution to MAE for (a) Cr, (b) S, and (c) Cl atom for the pristine CrSCl monolayer with spin orientation along (100) direction. Orbital-projected contribution to MAE for (d) Cr, (e) S, and (f) Cl atom for the hole-doped CrSCl monolayer with spin orientation along (100) direction.

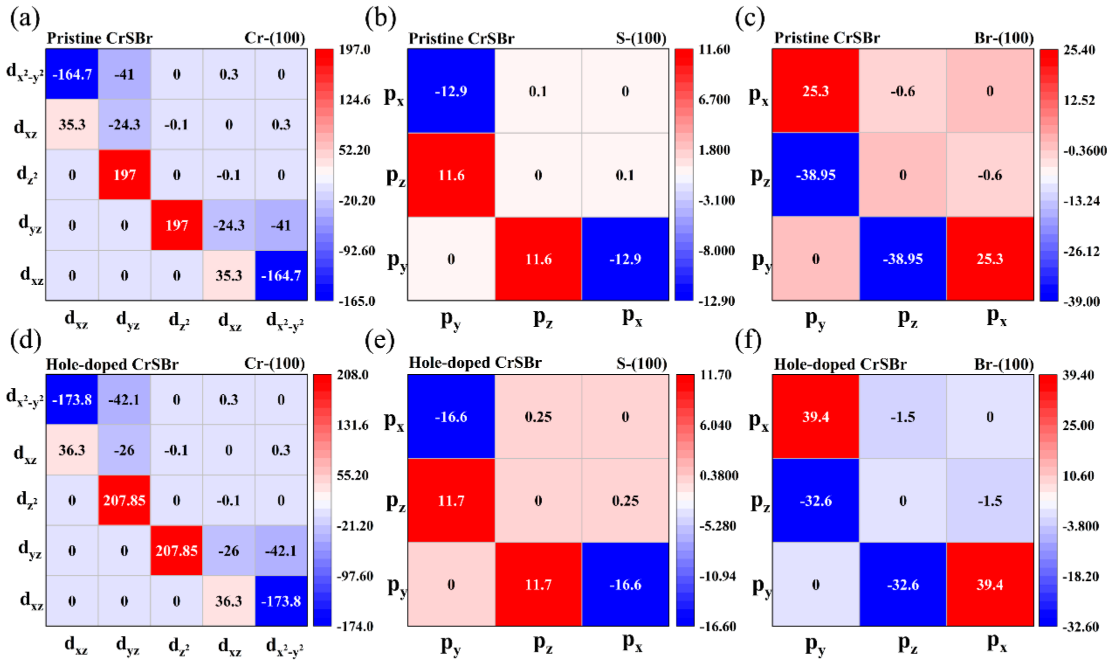

Figure S6. Orbital-projected contribution to MAE for (a) Cr, (b) S, and (c) Br atom for the pristine CrSBr monolayer with spin orientation along (100) direction. Orbital-projected contribution to MAE for (d) Cr, (e) S, and (f) Br atom for the hole-doped CrSBr monolayer with spin orientation along (100) direction.

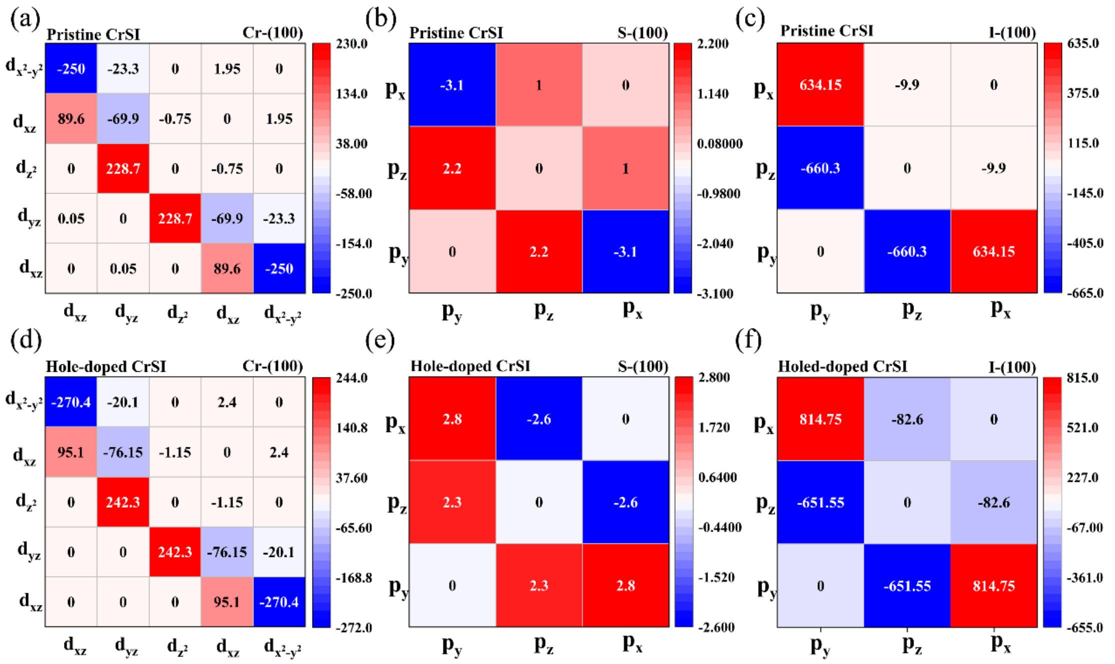

Figure S7. Orbital-projected contribution to MAE for (a) Cr, (b) S, and (c) Iatom for the pristine CrSCl monolayer with spin orientation along (100) direction. Orbital-projected contribution to MAE for (d) Cr, (e) S, and (f) I atom for the hole-doped CrSI monolayer with spin orientation along (100) direction.

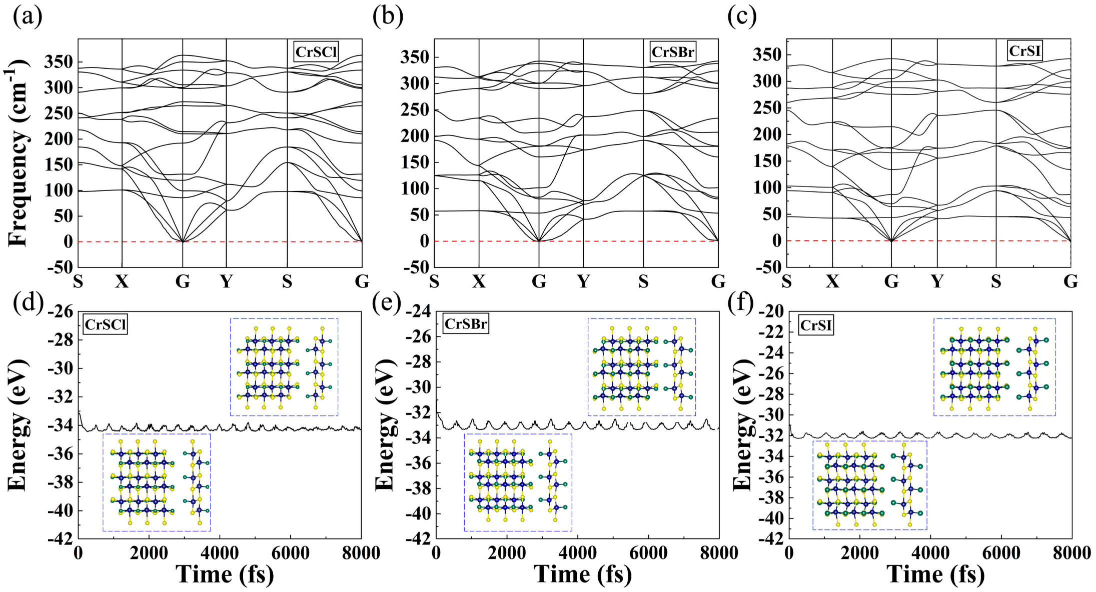

Figure S8. Phonon spectra of hole-doped 2D (a) CrSCl, (b) CrSBr, and (c) CrSI. AIMD simulations of hole-doped 2D (d) CrSCl, (e) CrSBr, and (f) CrSI at 300 K.
